# Advanced Applications of Porous Materials in Triboelectric Nanogenerator Self-Powered Sensors

**DOI:** 10.3390/s24123812

**Published:** 2024-06-13

**Authors:** Zhengyin Duan, Feng Cai, Yuxin Chen, Tianying Chen, Peng Lu

**Affiliations:** 1 Guangxi Key Laboratory of Clean Pulp & Papermaking and Pollution Control, College of Light Industry and Food Engineering, Guangxi University, Nanning 530004, China; 2316301011@st.gxu.edu.cn (Z.D.); 2016301002@st.gxu.edu.cn (F.C.); 2105170132@st.gxu.edu.cn (Y.C.); 2 National Engineering Laboratory of Textile Fiber Materials and Processing Technology, Zhejiang Sci-Tech University, Hangzhou 310018, China

**Keywords:** porous material, self-powered sensors, TENG, design strategies, structural forms

## Abstract

Porous materials possess advantages such as rich pore structures, a large surface area, low relative density, high specific strength, and good breathability. They have broad prospects in the development of a high-performance Triboelectric Nanogenerator (TENG) and self-powered sensing fields. This paper elaborates on the structural forms and construction methods of porous materials in existing TENG, including aerogels, foam sponges, electrospinning, 3D printing, and fabric structures. The research progress of porous materials in improving TENG performance is systematically summarized, with a focus on discussing design strategies of porous structures to enhance the TENG mechanical performance, frictional electrical performance, and environmental tolerance. The current applications of porous-material-based TENG in self-powered sensing such as pressure sensing, health monitoring, and human–machine interactions are introduced, and future development directions and challenges are discussed.

## 1. Introduction

**Figure 1:**
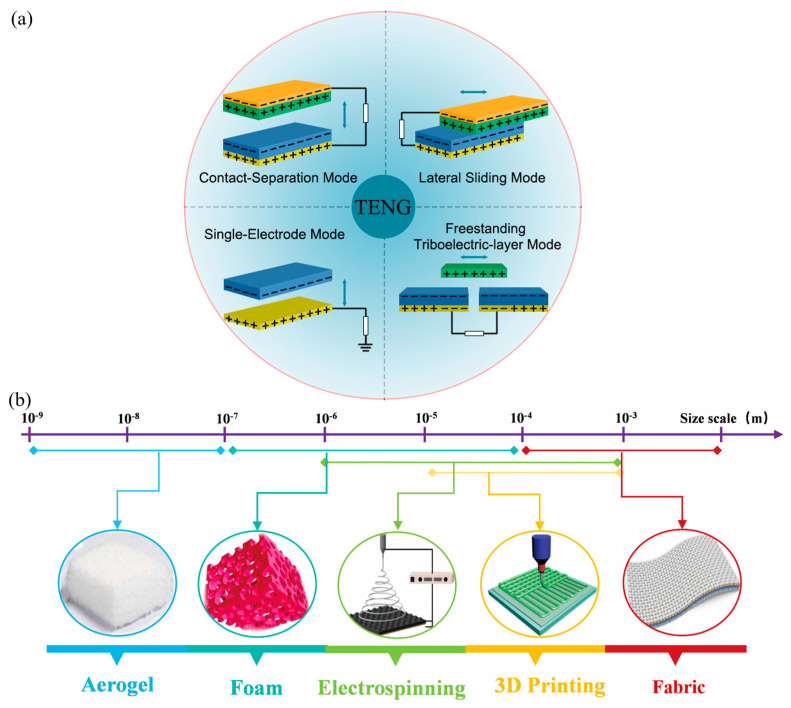
(**a**) Four typical operating modes of the TENG. (**b**) Various Porous Designs of TENG: Including Aerogels, Foam Sponges, electrospinning, 3D Printing, and Fabric.

With the rapid development of the Internet of Things (IoT) and artificial intelligence (AI) technology, there is an increasing demand for sensors with a small size, portability, and self-powering capabilities. To meet the requirements of large quantities and a wide distribution of applications, sensors need to have advantages such as being lightweight, portable, and having stable operation in different environmental conditions. A triboelectric Nanogenerator (TENG) is an energy-harvesting and conversion technology proposed based on the coupled effect of triboelectrification and electrostatic induction, which can harvest energy from the surrounding environment and achieve self-powered sensing. TENG has four typical operating modes, including a contact-separation mode, lateral sliding mode, single-electrode mode, and freestanding triboelectric-layer mode (Figure 1a). In these modes, electrons undergo a charge transfer between two electrodes through the contact and separation of triboelectric layers, resulting in the generation of an alternating current output [1]. TENG has advantages such as no need for an external power source, a small size, low cost, simple manufacturing process, and high sensitivity, making it one of the ideal choices for self-powered sensors. TENG has played an important role in various fields such as motion monitoring, biomedical, environmental management, and human–machine interactions. Using TENG as a sensing element requires high sensitivity in the electrical output performance, which poses special requirements on the structure and properties of materials. Porous materials, with their flexible and adjustable performance advantages, have increasingly important positions in the development of high-performance TENGs, especially in low-frequency energy harvesting and self-powered sensing, showing tremendous potential for applications.

Porous materials are defined as solids containing pores, typically with a pore volume fraction ranging from 0.2 to 0.95 [2]. Porous materials have advantages such as high porosity, a large surface area, low volume density, and low thermal conductivity, providing broad application prospects in adsorption and separation, catalyst support, thermal insulation, energy storage, sensors, and other fields. In the development of TENG self-powered sensors, designing frictional or electrode materials with porous structures can not only increase electrical signal output, but also offer advantages such as good breathability and being lightweight, providing various flexible solutions for expanding the functional applications of self-powered sensing.

In this paper, we provide a detailed introduction to the advanced application research progress of porous materials in TENG self-powered sensing, covering the forms and construction methods of porous structures, design strategies for enhancing the TENG performance of porous materials, and the current application status of TENG based on porous materials in pressure sensing, health monitoring, and the human–machine exchange. We also offer prospects for future development directions, aiming to provide inspiration for promoting the development and application of porous materials in the direction of TENG self-powered sensing.

## 2. Structure and Function

Porous materials feature interconnected porous networks with pore sizes varying from nanoscale to macroscale. According to the pore size, porous materials can be categorized into micropores (≤2 nm), mesopores (>2 nm and ≤50 nm), and macropores (>50 nm) [3]. The scale, morphology, and distribution of pores result in diverse mechanical properties and functionalities [4]. Macropores offer spacious cavities and transport channels, enhancing substance transfer, insulation, permeability, and deformability. Porous structures can also enhance energy absorption, playing a crucial role in improving the materials’ impact resistance and ability in large plastic deformation. The arrangement and distribution of porous characteristics influence their response to dynamic stress [5].

Porous materials with a high specific surface area and adjustable pore size distribution have played a crucial role in effectively enhancing the performance and multifunctionality of TENG [6]. In existing porous material-based TENG, various structural forms of porous materials are studied, with the most extensively researched five categories being aerogels, sponge foams, electrospinning membranes, three-dimensional (3D) printed porous structures, and fabrics (Figure 1b). These porous materials serve as friction layers or electrode layers, significantly improving the sensing performance of TENG [7], while greatly expanding their application potential in special scenarios, including antibacterial dust removal, noise reduction, thermal insulation, and electromagnetic shielding.

### 2.1. Aerogels

Aerogels are exquisitely porous structures crafted by replacing the liquid solvent in a gel with air, all without compromising the integrity of its solid network. Traditional methods for fabricating aerogels, such as solvent substitution, freeze-drying, and supercritical drying, while effective, have notable shortcomings, including substantial equipment costs and suboptimal yields. Fortunately, 3D printing technology has emerged as a cutting-edge alternative, enabling the production of customized aerogels tailored for applications in energy storage and sensing [8].

For the Aerogel-based TENG, the porous structure facilitates the formation of rough surfaces and provides a larger internal surface area, which helps generate additional charges during frictional electrical testing. On the other hand, the deformation of porous structures is much higher than that of dense films, facilitating a higher frictional electrical output. Highly porous and lightweight structures can reduce the weight of polymer materials used in TENG devices while providing breathability.

Aerogels can serve as frictional materials or electrodes in the structure of TENG. Among them, aerogels used as frictional materials include various materials such as polyvinylidene fluoride (PVDF), polyurethane (PU), polyimide (PI), and cellulose. Aerogels used as electrodes mainly consist of conductive materials such as porous carbon, MXene-doped aerogels, graphene oxide (GO), and carboxylated multi-walled carbon nanotube (CMWCNT) hybrid aerogels.

**Figure 2:**
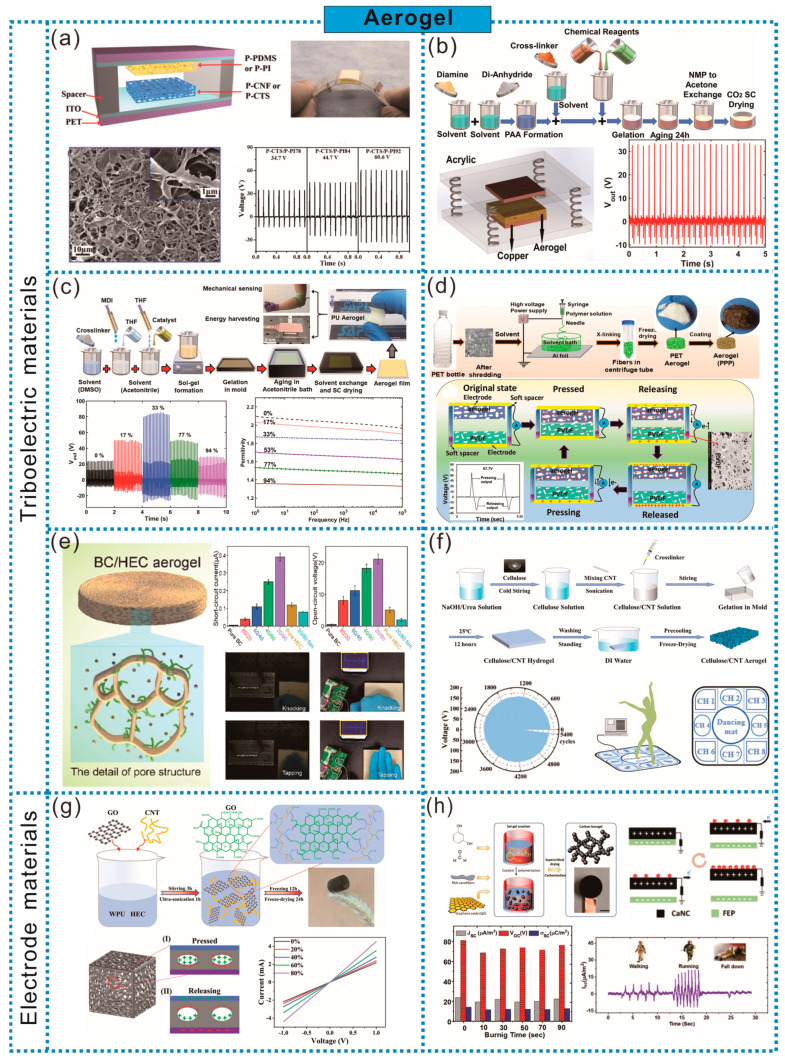
(**a**) Cellulose aerogel-based TENG [10]. (**b**) High-performance TENG based on porous PI aerogel film [11]. (**c**) PU aerogel-based TENG for high-performance energy harvesting and biomechanical sensing [12]. (**d**) Design of sustainable and flexible 3D aerogel using used PET bottles [13]. (**e**) Hybrid cellulose aerogel-reinforced TENG for energy harvesting and self-powered sensing hybrid cellulose aerogel-reinforced TENG [14]. (**f**) Regenerated cellulose-based TENG for dancer’s step sensing [15]. (**g**) Ultra-lightweight, elastic, hybrid aerogel for solid–solid/gas–solid TENG [16]. (**h**) Flame retardant, self-extinguishing TENG based on CaNC [17].

In recent years, the research on aerogels as frictional materials in TENG has attracted increasing attention. Compared to traditional dense film materials, the abundant porous structure of aerogels endows them with a higher specific surface area, which helps maintain a larger contact area during the frictional process, thus significantly improving the electrical output performance of TENG. For example, Wang et al. prepared PVDF aerogel films with a porosity as high as 97.9% by simple solvent exchange and freeze-drying. Compared to TENG based on non-aerogel PVDF, the performance was improved by 8.2 times, making it suitable for high-sensitivity self-powered motion sensors [9]. Additionally, under the same pressure stress, porous polymer films exhibit higher deformations compared to dense films, which increases the relative capacitance and thus enhances the frictional electrical output. As shown in Figure 2a, Gong et al. utilized chitosan (CTS) to prepare aerogel films via freeze-drying as the positive frictional material, which, combined with porous polydimethylsiloxane (P-PDMS), constituted aerogel TENGs (A-NGs). The frictional electrical output performance increased with the increasing porosity. Attributed to the increase in the contact area and electrostatic induction within the porous structure, the power output increased by up to 11 times under the same mechanical stress, compared to the corresponding dense film-based TENGs [10]. Saadatnia et al., based on p-phenylenediamine and biphenyl tetracarboxylic dianhydride, prepared PI aerogel films by the solvent exchange and CO_2_ supercritical drying of sol–gels. The aerogel film with a porosity of 50% significantly enhanced the performance of TENG, surpassing TENG with a simple PI layer by an order of magnitude. This enhancement was attributed to the increased effective surface area, the generation of additional charges on the internal porous surfaces of the aerogel, and the increase in the relative capacitance of the TENG device (Figure 2b) [11].

The increase in the porosity of aerogels leads to the addition of more air chambers in the sample, and an appropriate porosity can enhance the output performance of TENG. For example, as shown in Figure 2c, Saadatnia et al. obtained nanoscale porous PUA through sol–gel aging treatment and supercritical drying, and aerogel films with porosities ranging from 0 to 94% were obtained by adjusting the compression level. The presence of nanoscale pores not only improves the surface properties of aerogels, but also changes the capacitance behavior of TENG by altering the thickness of the aerogel. However, an excessively high porosity can reduce the material’s dielectric constant, resulting in a decreased TENG output. Therefore, it is necessary to select the appropriate porosity to the achieve optimal output performance. TENG composed of PUA films with an open porosity of 33% exhibited the highest performance, with an output voltage of up to 105.6 V and a peak current of 20.3 µA, which is 3.5 times that of TENG without pores. Additionally, due to the excellent flexibility of PUA aerogel films, PUA-TENG can be used to harvest energy during motion and for biomechanical sensing [12].

From the perspective of sustainable development, adopting recycled plastics or biodegradable materials to fabricate TENG embodies a more comprehensive concept of green energy. In terms of plastic recycling, Polyethylene terephthalate (PET) and Polystyrene (PS) account for a large volume of packaging waste, and aerogel materials prepared based on them exhibit relatively stable properties, meeting the application requirements of TENG well. For example, as shown in Figure 2d, Roy et al. utilized recycled PET plastic bottles as raw materials and, through electrospinning and freeze-drying processes, obtained soft, fluffy, and flexible PET aerogels. Subsequently, the surface modification of the fibers was performed using dopamine and PEI to form PPP aerogels. The higher surface polarity and electron-donating ability of PPP aerogels, with amino groups serving as excellent electron donors, result in higher frictional positivity. Additionally, the porous structure contributes significantly to the high output voltage and current. PPP-TENG can generate voltages and currents 18.2 times and 7.2 times higher, respectively, than those of uncoated PET A-NGs [13]. Moreover, aerogels prepared from materials such as cellulose and CTS have the advantage of natural biodegradability, making them more advantageous in achieving green and sustainable goals. As shown in Figure 2e, Lou et al. prepared a highly porous cellulose-based TENG using bacterial cellulose (BC) and hydroxyethyl cellulose (HEC) aerogels, which offers advantages such as biocompatibility and a low cost. Due to the higher surface roughness, lower surface potential, and highly porous structure, the TENG output performance was significantly improved, being more than 30 times that of pure BC aerogels and more than 80 times that of pore-free samples with the same HEC content. When BC/HEC aerogels are combined with wood boards, self-powered smart door panels can be obtained, converting mechanical energy from knocks into electrical energy to illuminate commercial light-emitting diodes (LEDs) or generate wireless signals on smartphone screens, helping the hearing-impaired identify knocks promptly [14]. Wang et al. dissolved microcrystalline cellulose in a pre-cooled NaOH/urea solution, added carbon nanotubes (CNTs) and crosslinking agents, cast it into hydrogels, and converted the hydrogels into regenerated cellulose carbon nanotube aerogels through washing and freeze-drying. The aerogel exhibited a 3D porous structure, high specific surface area, and enhanced dielectric constant, serving not only as a friction layer, but also as an electrode. Moreover, due to the dense structure of regenerated cellulose, it maintains an excellent output performance even at 99% humidity. The resulting TENG demonstrates an outstanding output performance, and when integrated into floor mats, can create self-powered dance mats, forming multi-channel human–machine interface sensors to evaluate the intensity and position of dancers’ steps (Figure 2f) [15].

In addition to serving as frictional electric materials, aerogels can also be used as electrode materials for TENG. The special design of the porous structure can endow the electrode with better compression strain, an enhanced electrical performance, lightweight flexibility advantages, and suitability for occasions with special requirements for high temperatures. For example, as shown in Figure 2g, Huang et al. added a certain amount of GO nanosheets and CMWCNT to a mixed solution of waterborne polyurethane and HEC, and prepared hybrid aerogels (GO/CNT HA) using the freeze-drying method, which were used as electrodes for TENG (GO/CNT HA-TENG), achieving high electrical output performance flexibility. The larger the compression strain of the aerogel electrode, the better the output performance of GO/CNT HA-TENG. This is because under large compression strain, in addition to the solid–solid contact electrifications (CE) coupling between the two charged layers, there is also a gas–solid CE coupling between the internal 3D skeleton of the aerogel electrode and the filled air. Compared with traditional TENGs, GO/CNT HA-TENG generates charge from 2D to 3D, and this unique advantage of 3D electrification promotes the enhancement of the final electrical output of GO/CNT HA-TENG [16]. Ahmed et al. prepared lightweight conductive mesoporous carbon aerogel nanocomposites (CaNC) based on the sol–gel polymerization of resorcinol–formaldehyde in the presence of polyacrylonitrile (PAN) nanofibers and GO nanosheets, followed by supercritical CO_2_ drying and high-temperature carbonization. The electrical, mechanical, and frictional electrical properties of the aerogel were improved due to the favorable charge transfer of PAN nanofibers and GO nanosheets. The aerogel can serve as both a frictional electric layer and an electrode without the need for the additional introduction of metal current collectors. Additionally, the aerogel also has flame-retardant and self-extinguishing properties. When paired with fluorinated ethylene propylene (FEP), a flame-retardant triboelectric nanogenerator forms and can generate a voltage of 80 V and a current of 25 μA/m^2^. The device exhibits excellent stability at temperatures above 200 °C and can be used as a tactile sensor for rescue tracking sensor systems [17] (Figure 2h).

### 2.2. Foam Sponge

Foam sponge is a material with abundant micrometer-to-millimeter-sized pores, featuring multiple advantages such as a wide range of raw material sources, simple processing techniques, and easy control over the pore size, morphology, and distribution. It is widely used in energy storage, sensors, photocatalysis, and other fields [18,19,20,21]. In the realm of TENG, foam sponge can be mainly categorized into conductive sponge and non-conductive sponge based on their functionalities. Conductive foam can consist of inherently conductive materials such as graphene foam or metallic foam, or it can be composite materials doped with conductive substances (e.g., conductive polymers like polypyrrole (PPy), metal particles, liquid metals, or CNTs). Non-conductive sponges are primarily made of elastic polymers, such as polydimethylsiloxane (PDMS), silicone rubber Ecoflex, polytetrafluoroethylene (PTFE), PU sponge, etc.

**Figure 3:**
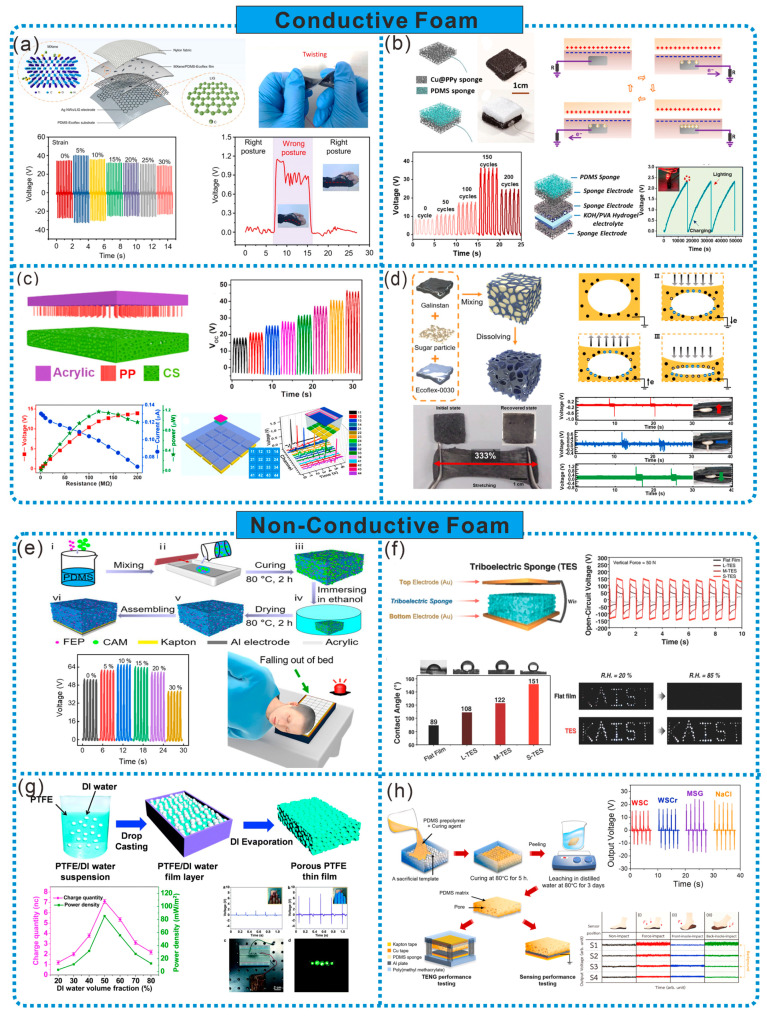
(**a**) TENG based on fully stretchable porous MXene–graphene foam nanocomposites [22]. (**b**) Bimodal TENG with sponge-like electrode brush structure [18]. (**c**) Liquid-metal-embedded sponge-like TENG for omnidirectionally detectable self-powered motion sensors [23]. (**d**) Multidirectional multi-amplitude TENG composed of porous conductive polymer [24]. (**e**) Smart pillow based on flexible breathable TENG arrays [26]. (**f**) Friction electric sponges with superhydrophobicity and elasticity through 3D soft lithography [27]. (**g**) S-TENG based on spongy porous PTFE film [28]. (**h**) Effect of pore morphology on mechanical and friction electrical properties of PDMS sponges prepared from commercially flavored templates [29].

Conductive foam can be used in TENG either as an electrode or simultaneously as both an electrode and friction layer. Compared to traditional electrodes, foam-structured electrodes exhibit improved flexibility and stretchability, and their rough surfaces can enhance the contact with the friction layer material. As shown in Figure 3a, Yang et al. employed a laser-induced method to generate graphene (LIG) foam in situ on the surface of PI film, where the highly porous 3D graphene network facilitated the penetration of the PDMS-Ecoflex prepolymer, resulting in the formation of LIG/PDMS-Ecoflex electrodes. By applying a pre-strain (with a pre-strain level of 30%) and spray-coating AgNWs solution, stretchable AgNWs/LIG electrodes were obtained after releasing the pre-strain. These LIG foam electrodes, together with porous MXene/PDMS-Ecoflex films, constituted friction negative friction layer, exhibiting high stretchability. It can be utilized as a self-powered strain sensor attached to clothing and skin surfaces for monitoring human activities and posture detection during strength training [22].

In addition to directly preparing conductive materials into conductive foam, another common method for producing conductive foam is by doping non-conductive foam with conductive substances. Composite materials not only possess the porous structure and large surface area of foam, but also offer a stable electrical output performance. As shown in Figure 3b, Li et al. treated PDMS sponge with vinyltrimethoxysilane silanization, followed by an ion exchange and chemical deposition, and finally deposited copper sponge by immersion in (NH4)_2_PdCl_4_. Further, the electrochemical deposition method was employed to transfer conductive PPy onto the Cu sponge, resulting in Cu@PPy sponge. This sponge exhibits excellent flexibility, durability, conductivity, and a large surface area-to-volume ratio, making it suitable for use as electrodes in TENG and flexible supercapacitors (SC). Moreover, a self-powered hybrid device was assembled using TENG and SC, providing prospects for integrated devices [18]. Introducing metal particles into polymer sponges is also a way to prepare conductive sponges. As shown in Figure 3c, Cui et al. prepared conductive sponge (CS) by covering nickel particles on a 3D PU network through chemical and electroplating processes. CS served as the positive friction material and was combined with polypropylene hairy elastic brushes as the negative friction material to form a TENG. Due to the interconnected porous network structure, CS exhibits high flexibility, elasticity, and surface area, enabling TENG to achieve a high electrical performance, excellent mechanical response sensitivity, and environmental adaptability in both contact-separation and sliding modes. The trajectory tracking sensor matrix prepared based on CS units demonstrates superior real-time monitoring, overall trajectory recording, and unique self-powered functionality, indicating the enormous potential of CS-based TENGs in self-powered trajectory tracking [23].

The porous structure of foam sponge not only increases the output power, but also enhances the tensile and compressive properties of the material. As shown in Figure 3d, a liquid-metal-embedded sponge-type TENG (LMST) with a special sponge shape was prepared using liquid metal (Galinstan) and silicone rubber (Ecoflex). LMST consists of randomly distributed pores and dispersed liquid metal droplets. LMST can bend 180° and stretch by 300%. Compared with the solid Ecoflex structure, the tensile and compressive properties are increased by 162% and 10%, respectively. Additionally, the shape of LMST can be flexibly designed using a mold, making the liquid metal embedded sponge TENG device flexible and suitable for a wide range of applications, such as detecting pressure, the direction of rotating balls, and the real-time detection of motor faults [24]. A flat-structured contact-separation TENG (F-TENG) has disadvantages of unstable output power and an inability to collect energy from multiple directions. These issues can be overcome by cleverly designing the structure of porous foam sponge. For example, Kim et al. mixed multi-walled carbon nanotubes (MWCNT) and PDMS, and then prepared porous conductive polymer (PCP) sponge structures using a sugar templating method. Subsequently, PCP-TENG was obtained by implanting a large number of coaxial aluminum wires coated with PTFE into PCP. PCP-TENG can effectively collect mechanical energy from various directions and amplitudes by utilizing the wide inner surface of the sponge structure as the contact area. Attached to the inner surface of a tire where F-TENG cannot be applied, LED lights were successfully illuminated with only the slight deformation of the tire, and the power was also supplied to a humidity sensor [25].

Non-conductive foam is primarily utilized as a frictional electric material in TENGs, enabling the design of multifunctional foams with excellent dielectric properties or mechanical performances by incorporating reinforcing materials, greatly advancing the development and application of high-performance TENGs. As shown in Figure 3e, Kou et al. utilized sacrificial templating to fabricate porous PDMS-containing FEP powder and assembled it into a flexible and breathable friction-based triboelectric nanogenerator (FB-TENG). By varying the porous structure and FEP powder content, the further optimization of the electrical output of FB-TENG was achieved. Ultimately, FB-TENG exhibited flexibility, comfortable breathability, outstanding stability, and high sensitivity. Intelligent pillows prepared based on this TENG can record real-time head movements during sleep and provide early warnings for emergencies [26]. Additionally, the unique manufacturing process and porous structure design of foam sponges can enhance the environmental tolerance of TENGs, expanding their applications under extreme conditions. For instance, as illustrated in Figure 3f, 3D soft lithography was employed to fabricate porous PDMS sponges assisted by sugar cube templates with different grain sizes, serving as a novel frictional electric sponge (TES). The unique micro/nanostructures in TES increase the effective contact area, thereby enhancing the electrical output performance. Under the same mechanical force, the power was increased by tenfold compared to flat PDMS membranes. Moreover, due to the upward force generated by air in the pores lifting water droplets, TES exhibits superhydrophobicity. Even in extremely humid environments (85% RH), it maintains a remarkably stable and high output performance, capable of lighting up 80 white LED lamps [27].

The porosity of foam sponges affects the output of TENGs, which can be optimized after adjustment for porosity. As shown in Figure 3g, Wang et al. used deionized water (DI) as a soft template to prepare sponge-like porous PTFE films, which were combined with an induction electrode to prepare a single-electrode TENG (S-TENG), and studied the effect of porosity on the output performance of S-TENG. When the volume fraction of DI was 50%, the output voltage was 1.8 times that of the solid PTFE film-based S-TENG under the same conditions. Meanwhile, the S-TENG has flexibility and a simple single-electrode structure, enabling it to collect mechanical energy from human motion [28]. Additionally, the pore morphology of foam sponges also affects their frictional electric performance and mechanical behavior. As shown in Figure 3h, Pharino et al. used different 3D geometric shapes of commercial seasoning (sugar cubes, sugar, monosodium glutamate, and NaCl crystals) as sacrificial templates to prepare PDMS sponges with different pore morphologies. It was found that PDMS sponges prepared using NaCl crystals as templates were the softest, with the lowest compressive modulus and a high frictional electric output. By incorporating poly (vinylidene fluoride-co-hexafluoropropylene) [P(VDF-HFP)] into the PDMS prepolymer and using NaCl as a template, composite sponges were prepared, further improving the electrical output performance. The obtained PDMS sponges were paired with aluminum plates to assemble TENGs for insoles, which can serve as self-powered foot pressure sensors to sense various human activities [29].

### 2.3. Electrospinning

Electrospinning is a versatile technique capable of producing 1D nanofibers (NFs), 2D nanofiber membranes, and complex 3D fiber structures [30]. Nanofiber membranes and 3D fiber structures prepared by electrospinning exhibit prominent features such as a high specific surface area, inherent rough structure, layered porous structure, and intrinsic porosity, which significantly enhance the surface charge density, flexibility, and breathability, making them highly suitable for frictional electric materials in TENGs [31]. Porous materials prepared by electrospinning are primarily used as the friction layer of TENGs with only a few as electrodes. The main materials include polyamide 66 (PA 66), PAN, PVDF, polycaprolactone (PCL), thermoplastic polyurethane (TPU), etc. In addition to directly using the above-mentioned materials for electrospinning, doping some nanofillers during electrospinning can enhance the frictional electric performance of electrospinning porous materials, such as metal–organic frameworks (MOFs), CNTs, etc. Moreover, controlling the parameters of electrospinning is also an effective method to improve the performance of frictional electric materials.

**Figure 4:**
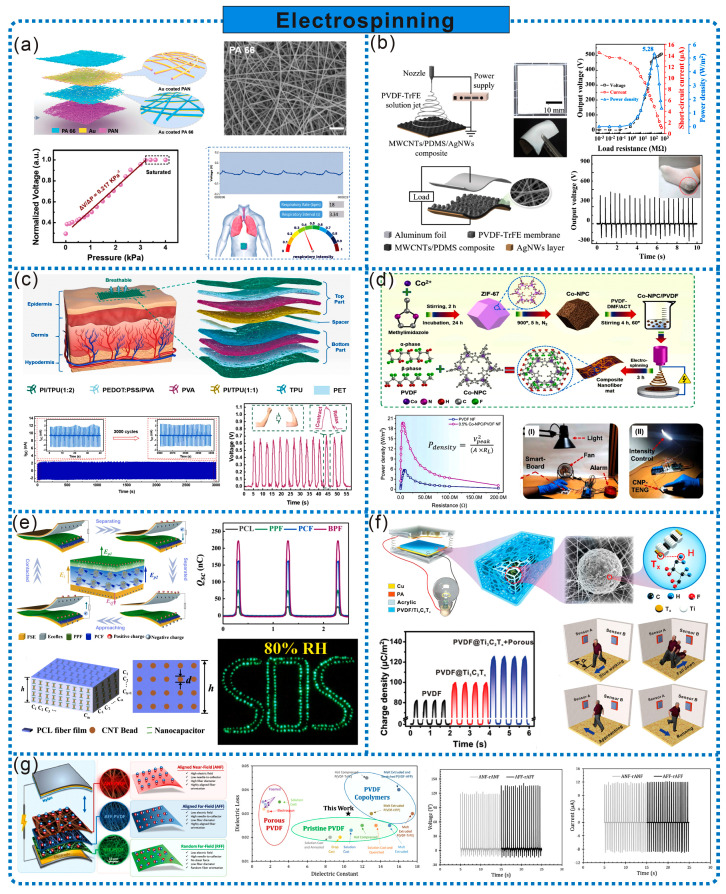
(**a**) Self-powered skin interface real-time respiratory monitoring system based on all-nanofiber TENG [32]. (**b**) Highly flexible TENG with electrostatically spun PVDF-TrFE nanofibers on MWCNTs/PDMS/AgNWs composite electrodes [33]. (**c**) All-nano/microfiber friction electric skin patch for physiological state monitoring [34]. (**d**) MOF-derived nanoporous carbon-bonded nanofibers for high-performance TENG and self-powered sensors [35]. (**e**) Asymmetric dielectric constant enhanced bilayer PCL nanofibers for high output and moisture-resistant TENG [36]. (**f**) Spherical multiphysics network friction electric materials for self-powered contactless sensing [37]. (**g**) High-performance TENG based on porous PVDF mats with significantly enhanced dielectric properties and novel dipole arrangement [38].

Electrospinning is a simple yet powerful technique for producing porous frictional electric materials for TENGs. By simple spinning and stacking, unique porous structures can be constructed, endowing the nanofiber membrane with excellent flexibility, breathability, and a good frictional electric performance. As shown in Figure 4a, Peng et al. developed a contact-separation-type TENG-based fully nanofiber electronic skin (SANES) using PA 66 and PAN nanofiber membranes obtained by electrospinning as the contact pairs and deposited gold as the electrodes. The multilayer stacked nanofiber network formed numerous 3D micro/nano multilevel porous structures, providing SANES with good breathability, while also enhancing the output power and pressure sensitivity (0.217 kPa^−1^). SANES can be attached to the abdomen, enabling the simultaneous autonomous energy and accurate real-time monitoring of subtle respiration [32]. Combining flexible electrospinning nanofiber membranes with flexible electrodes can further enhance the flexibility of TENGs. As illustrated in Figure 4b, Lee et al. fabricated a highly flexible TENG by electrospinning a polyvinylidene fluoride-trifluoroethylene (PVDF-TrFE) nanofiber membrane onto a MWCNT/PDMS/silver nanowires (AgNWs) composite electrode. Adjusting the PDMS mixing ratio can improve the bonding strength of the composite electrode, reduce the Young’s modulus, and further enhance the flexibility of TENGs. Due to the unique crystal structure of PVDF-TrFE, it can improve the output performance of TENGs (power density up to 5.28 W/m^2^). Additionally, TENGs can be used as power sources for flexible electronics and to harvest electrical energy from human motion [33]. Achieving the breathability of TENG electrodes is crucial for further improving the breathability of TENGs, and this issue can be addressed using electrospinning. As shown in Figure 4c, Qiu et al. manufactured a fully nanofiber/microfiber frictional electric skin patch (FNTOP) using a continuous electrospinning process. For the first time, a fully polymer-based electrode made of a poly(3,4-ethylenedioxythiophene):poly(styrene sulfonate)/polyvinyl alcohol(PVA) nanofiber membrane was used as the electrode, with a PI/TPU nanofiber membrane and TPU nanofiber/microfiber membrane as the frictional electric pairs. The fully polymer-based electrode can undergo direct electrospinning without the addition of any expensive metal materials or complex preparation processes. Due to the excellent breathability, hydrophobicity, and inherent flexibility of electrospinning membranes, FNTOP can be used for physiological status monitoring, such as interactive force detection, muscle movement analysis, respiration monitoring, finger gesture recognition, etc. [34].

Electrospinning technology is highly suitable for arranging nanofillers within electrospinning fibers [31]. Nanofillers can serve as oriented nuclei for polymer chains, thus imparting a higher surface charge density and mechanical stability to electrospinning fibers, which is advantageous for designing high-performance TENGs. MOFs possess excellent nanoporosity, a high surface area, and tunable pore size, making them suitable nanofillers for improving TENG performance. As shown in Figure 4d, Rahman et al. incorporated MOF-derived cobalt-based nanoporous carbon (Co-NPC) as the filler into a PVDF matrix, and then prepared Co-NPC/PVDF composite NFs through electrospinning to assemble into a TENG (CNP-TENG). The addition of Co-NPC significantly improved the formation of the electrically active β-phase and dielectric constant of the PVDF composite NFs, thus significantly enhancing TENG performance. Based on CNP-TENG, a self-powered pressure sensor was designed with ultra-high sensitivity of 6.39 V/kPa, suitable for various motion sensing and smart-home control system applications [35]. CNTs possess conductivity, and their introduction into NFs aids interface polarization and space charge accumulation. For instance, Zhong et al. incorporated CNTs into the interior of PCL fibers through electrospinning, preparing a bilayered PCL nanofiber felt (BPF) with integrated features such as a high specific surface area, strong hydrophobicity, and high dielectric constant, and combined it with a frictional negative silicone rubber to create a BPF-TENG. The introduction of CNTs resulted in a significant difference in the dielectric constant between the inner and outer PCL layers, endowing BPF with an excellent frictional electric performance. Due to the increased frictional area in the bilayer dielectric and dual interface polarization, the charge transfer of BPF-TENG increased by 740% compared to devices based on PCL gel films. Furthermore, the hydrophobic porous PCL nanofiber felt effectively reduces water droplet accumulation on the material surface, enabling BPF-TENG to continuously drive electronic devices to operate in humid weather conditions (Figure 4e) [36].

By adjusting the electrospinning solution and process parameters, NFs with a controllable morphology and structure can be obtained. Utilizing this method, various structural fiber membranes can be obtained without additional post-processing, providing a new solution for the development of high-charge-density frictional electric materials and high-performance TENGs. As shown in Figure 4f, Nie et al. prepared PVDF@Mxene (Ti_3_C_2_Tx) composite films with spherical multiple physical network structures by controlling the Rayleigh instability deformation and gas-phase separation of the electrospinning nozzle during the electrospinning process. With the presence of spherical multiple physical network structures and MXene (Ti_3_C_2_Tx), the composite film exhibits excellent chemical stability, a high specific surface area, high electronegativity, and good conductivity, capable of capturing and accumulating more negative charges, significantly enhancing the output performance and stability of TENGs. Self-powered non-contact sensors prepared based on this method demonstrate excellent speed sensitivity, accurately identifying motion states such as running, jumping, and walking within a range of 55 cm [37]. Additionally, controlling the dipole orientation technique through electrospinning is another method to improve the TENG output. As shown in Figure 4g, Rastegardoost et al. designed and prepared a porous PVDF felt pad by electrospinning. By arranging dipoles and introducing smart multilayer structures, the surface area of the PVDF felt pad was improved, overcoming the disadvantage of an insufficient dielectric performance caused by gaps in electrospinning pads. Using it as a frictional negative layer significantly improved the output performance of TENGs (an output voltage exceeding 130 V, currently up to 12 μA), which was significantly higher than pure PVDF and single-layer electrospinning pads [38].

### 2.4. Three-Dimensional Printing

Three-dimensional (3D) printing, also known as additive manufacturing, produces high-precision 3D products by adding liquid or solid materials layer by layer according to preset programs and digital models [39]. This technology is characterized by a low cost, fast molding speed, customized production, and the ability to manufacture complex structures without the need for templates, and it is widely used in many fields such as the automotive industry, healthcare, monitoring, and multifunctional wearable electronic products [40,41,42]. In the manufacturing of TENGs, the frictional electric layer, additional electrodes, system framework, and additional parts can all be completed in one step by 3D printing. Through 3D printing, it is easy to design the porous structure, porosity, and surface patterns of frictional electric materials, which can enhance the frictional electric performance and facilitate the production of high-performance TENGs. The main materials used for 3D printing include polyethylene (PE), nylon (PA), polyglycerol sebacate (PGS), PDMS, and cellulose.

**Figure 5:**
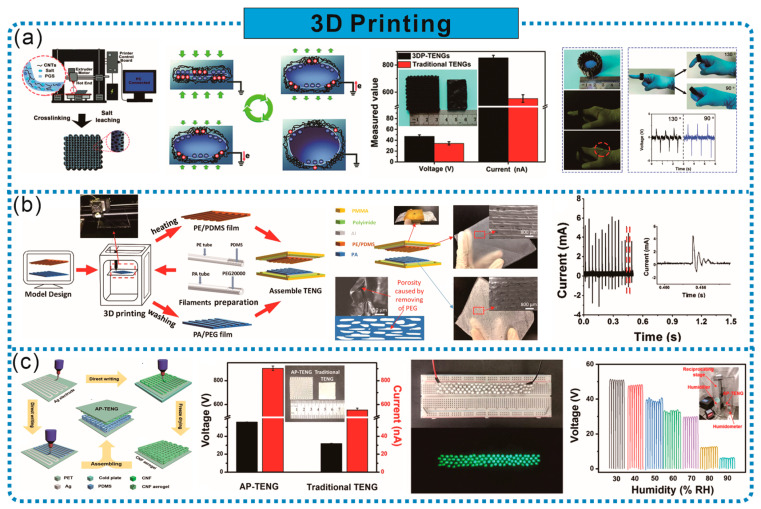
(**a**) Resilient, sustainable TENG based on a single integrated 3D printing process [43]. (**b**) Microstructured TENG based on 3D printing [44]. (**c**) Fully printed 3D hierarchical-structured cellulose aerogel-based TENG for multifunctional sensors [45].

Designing porous structures is an effective operation to enhance the output performance of TENG frictional electric materials, and 3D printing is well-suited for this task due to its advantages of speed, convenience, and the flexible design of pore shapes and sizes. As shown in Figure 5a, Chen et al. prepared CNTs and PGS in extrudable printing ink, and directly fabricated an elastic and sustainable TENG (3DP-TENG) in one step through 3D printing technology. This TENG features a multi-level porous structure, with each individual pore serving as a small TENG unit. Thanks to this 3D microstructure, the output voltage and current of 3DP-TENGs are superior to those of microhole TENGs manufactured by traditional molding methods. Leveraging the flexibility of 3D printing technology, 3DP-TENGs can be processed into various shapes (such as insole-shaped or ring-shaped) to meet different application scenarios [43]. Micro/nanostructures in frictional electric films can significantly improve the output performance of TENGs, and 3D printing technology combined with suitable materials can achieve the design of micro/nanostructures. As shown in Figure 5b, He et al. used tubular PE and PA as carriers, and directly printed PDMS and polyethylene glycol (PEG) filaments as the positive and negative friction layers of the TENG, respectively, without the need for templates through 3D printing. By removing PEG from the positive electrode film to form a sponge-like porous micro/nanostructure and designing different patterns directly through 3D printing, the electrical performance of the TENG was further improved [44].

In addition to designing porous structures, 3D printing can also enhance the frictional electrical performance of materials by designing surface 3D micro–nano multilevel patterned structures. As shown in Figure 5c, Qian et al. achieved this by printing cellulose nanofiber (CNF) ink and PDMS ink separately on Ag/PET substrates, forming 3D patterned positive friction layers and negative friction layers, thereby constructing cellulose aerogel-based triboelectric nanogenerators (AP-TENG) with 3D micro–nano multilevel patterned structures. The 3D patterns and nano-porous aerogel structure can significantly increase the device’s contact area, surface roughness, and mechanical elasticity, thereby aiding in improving the frictional electric response. Compared to their counterparts made through traditional molding techniques, AP-TENGs exhibit a higher voltage output, enabling the effective harvesting of mechanical energy to drive 88 LEDs and serve as self-powered mechanical and humidity sensors [45]. Jiang et al. summarized the roles of different 3D printing methods in the preparation of TENG components (frictional layers, electrodes, device frameworks, and other parts). These methods include Fused Deposition Modeling (FDM) technology, photopolymerization technology, and Direct Ink Writing (DIW) technology. Meanwhile, a series of TENGs with unique structures obtained using 3D printing for various energy harvesting and functional application scenarios were summarized. The perfect combination of 3D printing technology and TENG will provide a low-cost, simple, and efficient method for manufacturing energy harvesters and self-powered sensors, further expanding the functional applications of TENGs [39].

### 2.5. Fabric

Due to its ultrafine fiber scaffold structure, fabric can provide both micro and macro porous structures, high roughness, and a larger surface area, thus increasing the effective contact area and promoting the frictional electricity output. Fabric-based TENGs possess durability, flexibility, and breathability, making them primary candidates for biomechanical energy harvesting and wearable self-powered sensors. In TENGs, fabrics can be divided into two categories: non-conductive fabrics and conductive fabrics. In practice, these two are often used in combination, serving either as individual friction layers or electrodes, or simultaneously fulfilling the roles of both friction layers and electrodes. Non-conductive fabrics can be made of elastic polymers (such as PA, PVDF, etc.) or natural polymeric compounds (cotton fibers, cellulose). Conductive fabrics are typically made by doping conductive materials (metal nanowires, CNTs, PPy, etc.) into non-conductive fabrics.

**Figure 6:**
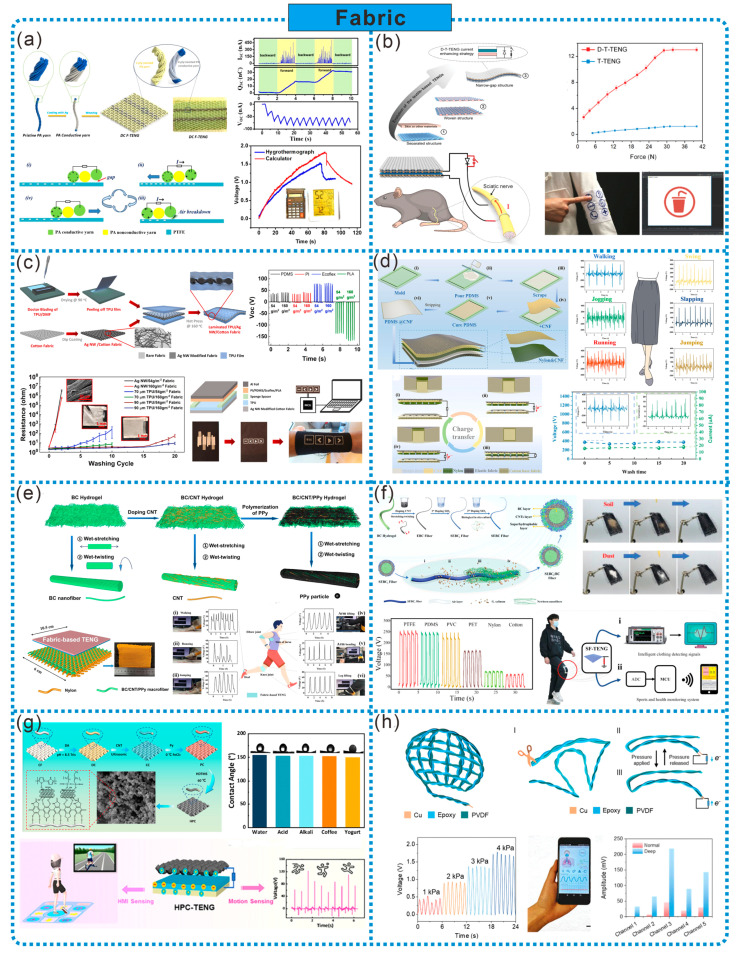
(**a**) DC fabric-based TENG for biomotor energy harvesting [46]. (**b**) Narrow-gap TENG fabric integrated with high-voltage diodes and fabric-based switches [47]. (**c**) Fabric wearable TENG for human–machine interface [48]. (**d**) Asymmetric elastic-structured fabric-based TENG for wearable energy harvesting and human motion sensing [49]. (**e**) Biodegradable, super-strong, and conductive cellulose macrofibers for fabric-based TENG [50]. (**f**) Fabric-based TENG woven with biofabricated superhydrophobic BC fibers for energy harvesting and motion detection [51]. (**g**) Moisture-resistant, conductive fabric-based TENG for efficient energy harvesting and human–computer interaction sensing [52]. (**h**) Deep learning-assisted mask sensor network for adaptive respiratory monitoring [53].

Fabric, as the most common porous material in our daily lives, has both micro and macro porous structures, a large surface area, and excellent flexibility and breathability, making it highly suitable as friction layer or electrode material for wearable TENGs. As shown in Figure 6a, Chen et al. used PA non-conductive yarns as warp yarns, and PA non-conductive yarns twisted with silver-coated PA conductive yarns as weft yarns, to design a plain weave structure direct-current fabric TENG (DC F-TENG). The separation of non-conductive and conductive yarns in the weft creates two electrodes (electrostatic breakdown yarn and friction yarn), allowing the DC F-TENG to cleverly harness the annoying and harmful phenomenon of electrostatic breakdown to collect kinetic energy and generate direct current electricity. Weaving solid-state yarn SC into the DC TENG fabric as a wearable whole enables energy harvesting and storage, providing power for electronic devices such as thermometers and calculators [46]. Softness and thinness are the main features of fabric-based TENGs compared to other types of TENGs, and it is essential to enhance the output of fabric-based TENGs without compromising these features. Therefore, He et al. attached a thin and wrinkled layer of nitrile film to conductive fabric as the positive friction material, coated another conductive fabric with silicone rubber as the negative friction material, stitched them face-to-face with a narrow gap, and sealed them with non-conductive fabric to create a narrow-gap TENG fabric. Then, by integrating it with high-voltage diodes and mechanical switches, they prepared a diode-enhanced fabric-based TENG (D-T-TENG). Compared to pure fabric-based TENGs, the closed-loop current of D-T-TENGs can be increased by 25 times. D-T-TENGs have the characteristics of softness and thinness, and can achieve a moderate output even with random rubbing. Additionally, the enhanced output current of D-T-TENGs can stimulate muscles and nerves, achieving controlled muscle stimulation. They can also be embedded in clothing to power humidity and temperature sensors [47] (Figure 6b).

Improving the washability of fabrics can effectively expand the application scenarios of TENG sensors. As shown in Figure 6c, Doganay et al. laminated a TPU film onto Ag NW-modified cotton fabric as the bottom electrode and PLA/Al foil as the top electrode to form a TENG. This fabric-based TENG maintains good durability, heating behavior, and uniformity, enduring 15 washing cycles. A self-powered electronic wristband developed based on this TENG can serve as a human–computer interface for controlling simple computer operations [48]. Additionally, Gao et al. fabricated an asymmetric elastic structural fabric-based TENG (AesF-TENG) using stretchable elastic fabric and conventional cotton fabric as the substrates, and nylon and PDMS doped with barium titanate nanoparticles as the positive and negative triboelectric materials, respectively. Due to the elasticity difference between the two fabrics, AesF-TENG can achieve contact-separation processes under minimal deformation or force, better matching various human movement patterns. Sewing it onto clothing effectively collects biomechanical energy and serves as a self-powered sensor for detecting human motion. Moreover, after 20 washes and 120,000 cycles of testing, AesF-TENG still efficiently collects biomechanical energy and maintains a stable electrical performance [49] (Figure 6d).

As the concept of sustainability becomes increasingly prevalent, the development of environmentally friendly fabric-based TENGs aligns more closely with current and future requirements. Cellulose, as a natural polymer, possesses abundant resources, environmental friendliness, and renewable characteristics, offering significant advantages in green sustainability. As shown in Figure 6e, Hu et al. incorporated CNTs and PPy into BC, and then utilized wet stretching and wet twisting methods to prepare a super-strong, biodegradable and washable cellulose-based conductive large fiber. This fiber exhibits a high tensile strength of 449 MPa, good conductivity (~5.32 S cm^−1^), and excellent stability. Fabric-based TENGs prepared from this fiber can be attached to various parts of the human body to serve as self-powered sensors for detecting human motion [50]. As depicted in Figure 6f, Chen et al. doped CNTs into the surface of BC hydrogels, obtained conductive BC fibers through wet twisting processes, then modified them with hydrophobic SiO_2_ nanoparticles and formed a shell structure through biomineralization, ultimately producing superhydrophobic conductive BC fibers (SEBC fibers). Fabric-based TENGs (SF-TENGs) can maintain a stable output performance even under harsh environmental conditions such as liquid spillage. Smart clothing and sports health monitoring systems designed based on SF-TENGs can monitor physical activities in various scenarios and record movement intensity and frequency [51].

Fabric can serve either as a friction layer or electrode in TENG, or it can serve as both the friction layer and electrode simultaneously. As illustrated in Figure 6g, He et al. prepared a superhydrophobic conductive composite fabric (HPC) by loading polydopamine (PDA), CNTs, PPy, and hexadecyltrimethoxysilane onto the surface of cotton fabric. They then used this fabric as both the friction positive layer and electrode layer to manufacture a TENG (HPC-TENG). The synergistic enhancement of conductivity in the composite fabric was achieved through hydrogen bonding between PPy and CNTs. Additionally, the electron-donating effect of amino groups in PPy further enhanced the positive polarity of the fabric’s friction. Compared to unmodified fabric-based TENGs, the HPC-TENG exhibited a nearly 7.2-fold improvement in performance. Furthermore, the HPC-TENG can be used as a pressure sensor for monitoring human motion states and as a multi-channel sensor for smart game blankets [52].

Incorporating sensing and therapeutic capabilities into everyday fabric is an effective method for developing personalized healthcare. For example, inspired by cribellate silk, Fang et al. used PVDF and alloy wire as raw materials to induce spindle-knotted fiber structures using Rayleigh instability, which were then woven into permeable and moisture-resistant fabric triboelectric sensors. An intelligent mask sensor network for respiratory monitoring was constructed based on computational fluid dynamics simulations and deep learning, which can overcome interference from different user facial contours and environments, accurately identifying respiratory signals. Integration with a smartphone app enables the real-time data-driven diagnosis and one-click sharing of health data with doctors [53] (Figure 6h). Furthermore, Chen et al. reviewed the development of smart fabric in the field of personalized healthcare. They investigated various platform technologies, manufacturing strategies, and clinical scenarios for the use of smart fabric, exploring their applications in diagnosis and treatment. This provides guidance for the development of multifunctional smart fabric for medical and healthcare applications [54].

## 3. Performance Enhancement Strategies

The pursuit of high-performance TENGs is crucial for their advanced applications, encompassing not just the electrical, output but also the robust mechanical performance, stability, and resistance to environmental factors. Achieving these comprehensive properties remains challenging, often requiring trade-offs. Recent research suggests that designing porous materials with unique structures or incorporating other functional materials into composite porous structures offers promise in elevating the overall performance of TENGs. Specifically, we are exploring how to optimize their sensing capabilities in terms of their mechanical robustness, triboelectric performance, gas responsiveness, and environmental tolerance.

### 3.1. Mechanical Performance

An excellent mechanical performance is essential to ensure the sensing performance and normal application of porous material-based TENGs. Having certain outstanding mechanical properties, such as high compressibility, high strength, low modulus, and superelasticity, can enable TENGs to be applied in special fields and environments, or enhance the sensitivity and detection range of TENG-based sensors. Therefore, improving the mechanical performance of porous materials is an indispensable aspect of preparing high-performance TENGs. Sensors based on TENGs have been evolving towards being lightweight and prioritizing portability. However, achieving the excellent mechanical performance of porous materials at a low density remains a challenge. Leveraging the characteristics of porous materials, we can achieve their outstanding mechanical performance through unique structural designs.

**Figure 7:**
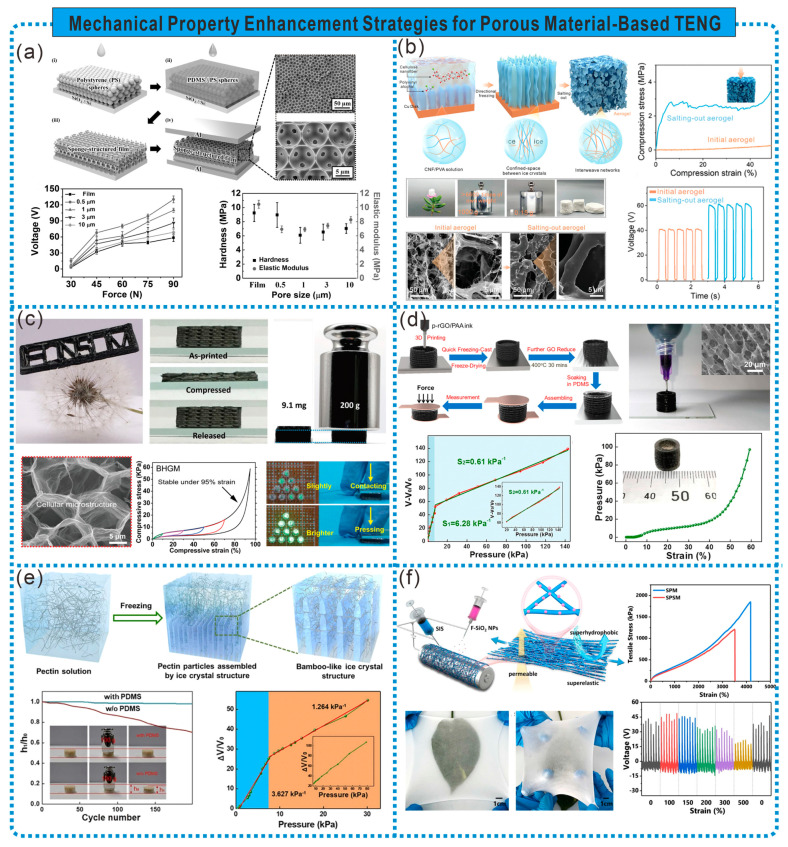
(**a**) TENG based on hydrophobic sponge structure [55]. (**b**) Hoffmeister effect-induced multiscale-structured nanocellulose friction electrogel [56]. (**c**) Ultralight biomimetic multistage graphene material with excellent stiffness and elasticity [57]. (**d**) Self-powered friction electric pressure sensor based on 3D-printed endoplasmic reticulum rGO microstructure [58]. (**e**) Bamboo-inspired self-powered friction electric sensor for touch sensing and sitting posture monitoring [59]. (**f**) Silk-inspired in situ interlocking superelastic–hyperelastic microfibers for permeable stretchable TENG [60].

The construction of nanoporous structures can endow materials with higher compressibility, and can change their capacitance under stress to enhance the output of TENGs. As shown in Figure 7a, Lee et al. assembled a sponge-structured TENG (S-TENG) using highly compressible mesoporous structured PDMS films and aluminum electrodes. Compared to flat films, sponge-structured films exhibit a higher specific surface area and compressibility (over 30% decrease in the elastic modulus). Under the same mechanical force, the distance between the two electrodes in sponge-structured films is smaller, with a larger dielectric constant and contact area. Consequently, compared to flat-film TENGs, S-TENGs show a tenfold increase in the output power, which increases with the decreasing pore diameter. Furthermore, benefiting from the micro–nano porous structure, S-TENGs demonstrate a stable power output performance even at 80% relative humidity [55]. The shrinkage and collapse of porous structures can severely affect the stability and accuracy of frictional electric sensor responses. The preparation of porous structures with stiffness is conducive to improving the stability of frictional electric material outputs. As shown in Figure 7b, Luo et al. fabricated rigid nanofibrillar frictional electric hydrogels with multiscale structures using a combination of the Hofmeister effect and freeze-drying. Due to the Hofmeister effect altering the internal network structure of the polymer, enhancing polymer chain aggregation and crystallization, the hydrogel exhibits excellent stiffness with a Young’s modulus of up to 142.9 MPa. Even after enduring an impact of 343 kPa, wearable self-powered sensors made from this hydrogel can still work [56].

Natural biomaterials assemble complex hierarchical structures from micro to macro scales, achieving excellent mechanical performances at low densities. This inspires researchers to design and manufacture porous materials with outstanding mechanical properties. Ink-based 3D printing strategies offer advantages in designing 3D structures from micro to macro scales and easily controlling macroscopic geometries and sizes, making them suitable for this work. As shown in Figure 7c, Peng et al., inspired by Elytrigia repens, based on 3D printing strategies and optimized partially reduced GO ink, combined with freeze-casting treatment, constructed a 3D graphene material with macroscopic hollow scaffolds and microstructural biomimetic layered structures (BHGM). Benefiting from the macroscopic and microstructural biomimetic layered structure, BHGM exhibits excellent stiffness, elasticity, and ultralightness. BHGM’s Young’s modulus is over three times higher than that of traditional elastic graphene materials and shows outstanding elasticity at an ultra-low density (8.5 mg cm^−3^). TENGs assembled with BHGM as an elastic deformation electrode can generate a large voltage under low-frequency compression [57]. Additionally, as shown in Figure 7d, Lei et al. fabricated PDMS@rGO pads and dielectric layers with hollow ring macrostructures and inner mesh rGO microstructures using 3D printing and subsequent freeze casting, assembled with copper foil electrodes to form a self-powered frictional voltage pressure sensor (rGO-TPS). Due to the ultra-low Young’s modulus and special structural design of 3D printing materials, rGO-TPS exhibits high sensitivity and a wider high-sensitivity range. rGO-TPS can monitor dynamic pressure responses by detecting changes in pulse-like short-circuit current signals, used for measuring water droplets, airflow, and vibration signal identification [58]. The sensitivity and detection range of frictional voltage sensors under high pressure can also be optimized by designing the internal structure of the dielectric layer. For example, inspired by bamboo, Lei et al. fabricated a dielectric layer with disc-like macrostructures and bamboo-joint microstructures using 3D printing and freeze-drying and, based on this, prepared a self-powered frictional electric sensor (BSTS). The hollow macrostructure provides a lower effective stiffness coefficient, aiding in the large compressibility of the dielectric layer, while the bamboo-joint microstructure acts as a reinforcement film, enhancing the toughness of the dielectric layer. Thanks to this unique structure, BSTS can effectively balance high sensitivity and a wide detection range. It can be used for controlling external circuits like lighting systems and the real-time monitoring of posture [59] (Figure 7e).

Using electrospinning to achieve the in situ welding of conductive materials on fibers can achieve mechanical/electrical robustness. As shown in Figure 7f, Li et al. achieved clever in situ welding effects at partially cured fiber joints by synchronously electrospinning styrene–isoprene–styrene block copolymers and electrostatically spraying fluorinated SiO_2_ nanoparticles (F-SiO_2_NPs) onto fibers. Meanwhile, the deposited F-SiO_2_NPs were embedded in the fibers, preparing fiber membranes with omnidirectional super elasticity (with an ultimate elongation exceeding 3600%), permeability (over 90% cotton), and superhydrophobicity (WCA of 154.2°) (SPSM). Furthermore, based on the solvent-induced anchoring effect between SPSM and the conductors, adjustable conductivity and electrical robustness were designed for permeable elastic electrodes. Self-cleaning single-electrode stretchable TENGs (STENGs) based on SPSM can adapt to tapping, stretching, bending, and humid environments, suitable for breathable self-powered sensors for material identification and hand gesture monitoring [60].

### 3.2. Electrical Performance

An excellent frictional electrical performance is the key to achieving the outstanding sensing performance of TENG-based sensors. Chemical modification to enhance the frictional polarity of porous materials has been recognized as an effective method to enhance the electrical performance of TENGs [61]. Designing unique pore structures through physical methods, incorporating nanoparticles with high dielectric constants, the value of ε/d can also enhance the frictional electrical performance.

**Figure 8:**
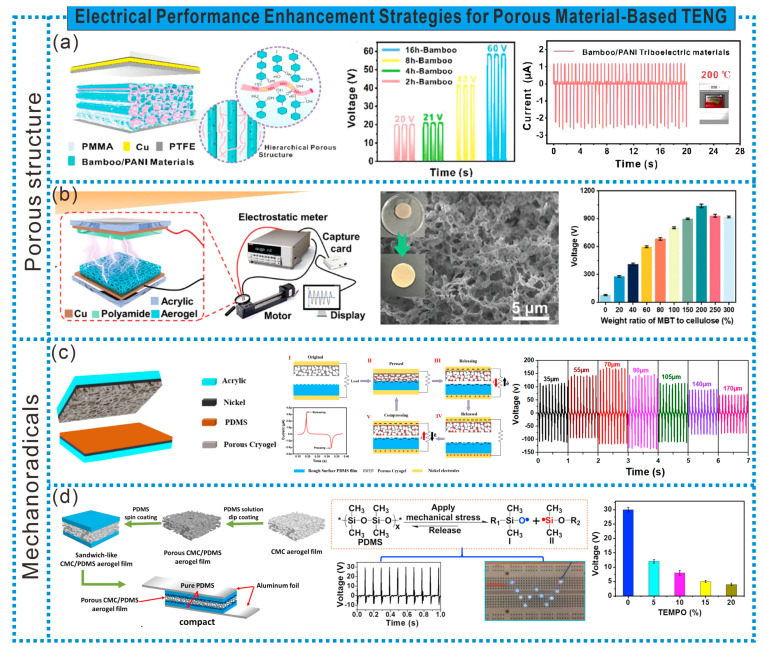
(**a**) Multi-stage porous cellulose friction electric materials suitable for extreme environmental conditions [62]. (**b**) Cellulose-based composites with excellent negative friction electric properties [63]. (**c**) Highly porous polymer low-temperature-based friction-positive materials for high performance TENG [64]. (**d**) Novel flexible TENG consisting of porous aerogel films driven by mechanical radicals [65].

The unique 3D interconnected porous structure of porous materials can not only provide a large specific surface area, generating more surface charges, but also reduce the thickness of the porous materials under stress, thereby increasing the capacitance and improving the electrical output of TENGs. As shown in Figure 8a, Zhao et al. first treated bamboo with a hydrogen peroxide hydrolysis system, supplemented by freeze-drying treatment, to form many different-sized multi-channel pores on the surface and within the wall cavity of bamboo. Then, they impregnated the bamboo with aniline monomers using a liquid phase transformation method, forming a multi-level porous bamboo/polyaniline (PANI) frictional electric material (BPTM) with continuous conductive pathways. The special multi-level porous structure endows the BPTM with a larger contact area and more induced charges, thereby enhancing the electrical output performance of the TENG, surpassing most wood fiber-based frictional electric materials. Additionally, thanks to the mutual protection between the cellulose scaffold and internal electrodes, BPTM-based TENGs can be used for self-powered sensing in extreme environments (high temperature, low temperature, or repeated thermal shocks) [62]. Furthermore, Wang et al. prepared modified cellulose aerogels with abundant mesopores and a complex network structure by dissolving and regenerating cellulose and incorporating trimethoxy(1H,1H,2H,2H-perfluorodecyl)silane (THS). Barium titanate (MBT) and PVDF were then added to prepare cellulose-based composite materials with an excellent negative frictional electrical performance. The addition of THS reduced the surface free energy of cellulose, increasing the frictional polarity. Moreover, THS anchored the high dielectric MBT on the cellulose surface, making the internal channels of the composite material more complex, with a stronger electron capture capability and attraction, thereby greatly improving the electrical characteristics of cellulose-based TENGs. The output voltage can reach 1040 V, with a current of 1.165 mA, superior to all cellulose-based negative frictional electric materials (Figure 8b) [63].

Research has found that the generation of mechanical free radicals in porous structures can further improve the electrical output performance of TENGs. As shown in Figure 8c, Haider et al. prepared high-porosity cryogel films based on lauryl methacrylate-acrylamide (LA-AAm) using a simple combination of freeze–thaw, ultraviolet radiation, and thawing processes. The high-density pores in the cryogel film can provide a larger surface area and generate a high density of mechanical free radicals, significantly improving the TENG’s output performance [64]. Additionally, the generation of mechanical free radicals enables TENGs to achieve a high electrical output without using traditional piezoelectric materials or air gaps. As shown in Figure 8d, Tang et al. coated a thin layer of PDMS on the surface of a compressed porous CMC aerogel film, then sandwiched it between two layers of thin PDMS film, and finally sandwiched it between two aluminum foils to prepare a flexible, gap-free TENG. Due to the reversible and transient dipole moments generated by mechanical free radicals in the PDMS film and the permanent dipole moments exhibited by the polarity groups induced by mechanical free radicals, the TENG demonstrates an excellent electrical output performance. The open-circuit voltage and short-circuit current are 30.0 V and 4.5 μA, respectively, corresponding to a power density of 1.1 W/m^2^. This indicates that flexible porous polymer films capable of efficiently generating mechanical free radicals can be prepared without polarization and air gaps to achieve high-output flexible TENGs [65].

**Figure 9:**
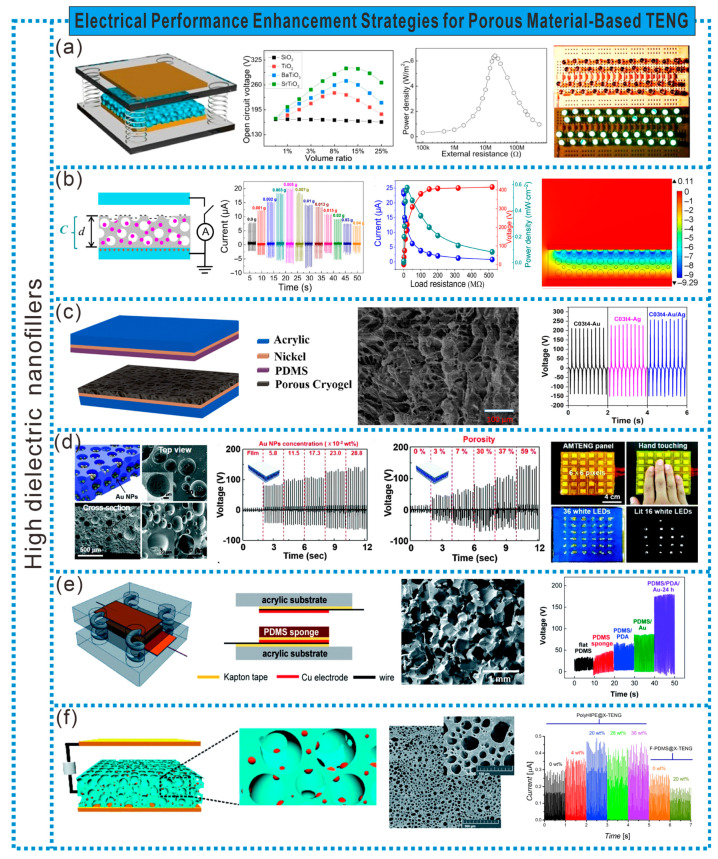
(**a**) Enhancement of TENG by filling high-dielectric nanoparticles into sponge PDMS film [66]. (**b**) Variable microcapacitor embedded in PDMS to improve the output power of TENG [67]. (**c**) Highly porous and thermally stable friction-positive hybrid bimetallic cryocrystalline gel improves the performance of TENG [68]. (**d**) Mesoporous PDMS films impregnated with gold nanoparticles as an effective dielectric to improve the performance of TENG in harsh environments [69]. (**e**) Dielectric control of porous PDMS elastomers with Au nanoparticles to enhance the output performance of TENG [70]. (**f**) PDMS nanocomposite macroporous film prepared by Pickering high-internal-phase emulsion as an effective dielectric to improve the performance of TENG [71].

The charge density on frictional electric materials is closely related to their capacitance, and it can be better designed by simultaneously introducing pores and nanoparticles with high dielectric constants into the frictional electric materials to modify ε/d values. Therefore, filling high dielectric nanoparticles into the pores of porous materials is an effective method to improve the TENG’s electrical performance. As shown in Figure 9a, Chen et al. prepared a high dielectric PDMS composite film by filling high dielectric nanoparticles [SiO_2_ (dielectric constant εr = 3), TiO_2_ (εr = 80), BaTiO_3_ (εr = 150), SrTiO_3_ (εr = 300)] into a porous PDMS sponge film. It was used as the friction layer with copper serving as another friction layer and electrode to form a high-output-performance TENG. Using a film composed of 10% SrTiO_3_ nanoparticles and 15% pores, the power was increased by more than five times compared to the TENG based on a pure PDMS film, easily lighting up 44 series-connected LEDs [66]. Metal nanoparticles in the pores of porous materials form micro-capacitors, enhancing the electrical output of the frictional electric materials. As shown in Figure 9b, by filling silver nanoparticles into PDMS and constructing internal honeycomb structures, micro-capacitors (MCs) and variable micro-capacitors (VMCs) were embedded in PDMS to prepare an MCs@PDMS film and VMCs@PDMS film as two types of frictional electric materials. The micro-capacitors formed by Ag NPs increase the effective capacitance, enhancing the accumulation of surface charge on PDMS. Due to the presence of variable micro-capacitors, the output power of TENGs based on a VMCs@PDMS film can be controlled when subjected to external forces. Moreover, the output current of TENGs based on a VMCs@PDMS film is four times higher than that based on a pure PDMS film, and 1.6 times higher than that based on an MCs@PDMS film [67]. Additionally, filling high dielectric metal nanoparticles can also enhance the positive polarity of frictional materials, and introducing multiple metal nanoparticles simultaneously is more beneficial for the electrical output of TENGs. For example, Haleem et al. prepared silver and gold nanoparticles using an in situ reduction reaction on UV-radiation cryopolymerized P(LA-AAm) and finally obtained a hybrid cryogel, P(LA-AAm), embedded with silver and gold nanoparticles, which was freeze-dried to obtain a porous hybrid bimetallic cryogel film. The porous and rough structure, as well as the interaction of metal nanoparticles, enhanced the output performance of the frictional positive electrode material, all higher than those of the TENG composed of pure cryogel [68] (Figure 9c). This bimetallic hybrid cryogel provides a new strategy for enhancing the performance of frictional positive materials and designing high-performance TENG devices.

Filling high-dielectric nanoparticles while controlling the porosity of porous materials is also crucial for enhancing the TENG’s electrical performance. As shown in Figure 9d, Chun et al. prepared block-like PDMS films with mesoporous structures by casting a mixture of PDMS solution and DI and then slow-drying. Au NPs were subsequently deposited at the bottom of the mesoporous PDMS film and attached between two PDMS film/PI/Al electrodes to create a seamless TENG (AMTENG). The increase in the charge density generated by the contact between Au NPs and PDMS within the pores enhanced the electrical output performance of the TENG. Compared to a flat-film TENG under the same cyclic compressive force, the power was enhanced by more than five times. Moreover, the electrical output performance of AMTENG significantly improved with an increase in porosity. Additionally, AMTENG can be used for self-powered shape-mapping sensors [69]. Furthermore, evenly attaching metal nanoparticles to the inner surface of pores is more conducive to increasing the charge density within the pores compared to deposition only at the bottom of the pores. As shown in Figure 9e, Biutty et al. used a sugar template method to prepare PDMS sponges with 3D interconnected pores. PDMS was then sequentially immersed in a PDA solution and Au NP water solution to prepare PDMS/PDA/Au composite materials. With the introduction of Au NPs, the dielectric constant of the composite material increased to 3.70 (mesoporous PDMS sponge was 2.94). Moreover, the presence of the PDA layer enabled the uniform adsorption of Au NPs on the surface inside the PDMS pores. During compression, the local contact between Au NPs and the PDA layer effectively transferred charges from Au NPs to the pore surface, significantly increasing the charge density within the pores. The average open-circuit voltage of the TENG reached 180 V, approximately 5.5 times higher than that of flat PDMS [70].

Additionally, preparing nano-composite porous friction materials using Pickering high-internal-phase emulsion (HIPE) template technology provides a novel, effective, and simple method for enhancing the TENG’s performance. For instance, Flores et al. employed HIPE template technology with commercial PDMS and distilled water as the continuous phase and dispersed phase, respectively. A mixture of surfactant (Span 20) and Ag NPs was used as the emulsion stabilizer to prepare PDMS nano-composite macroporous films (PDMS-NC), which were then assembled with Cu as the negative friction material for the TENG. The PDMS-NC film exhibited an interconnected 3D macroporous structure with Ag NPs on its porous surface. The Ag NPs not only enhanced the emulsion stability, but also formed a capacitor structure to improve the material’s dielectric performance. The resulting TENG has a power increase of over 1.3 times compared to PDMS films without porosity or Ag NPs [71] (Figure 9f).

### 3.3. Gas Responsiveness Characteristics

The unique porous structure of porous materials gives them a high specific surface area and porosity, which can significantly increase the binding sites for gas molecules. Meanwhile, the introduction of certain special functional materials [such as CNTs, Ti_3_C_2_Tx, PANI] can also enhance the mechanical, electrical conductivity, and gas responsiveness performance of porous materials. This lays the foundation for the design of self-powered gas-sensitive smart sensors based on TENG.

**Figure 10:**
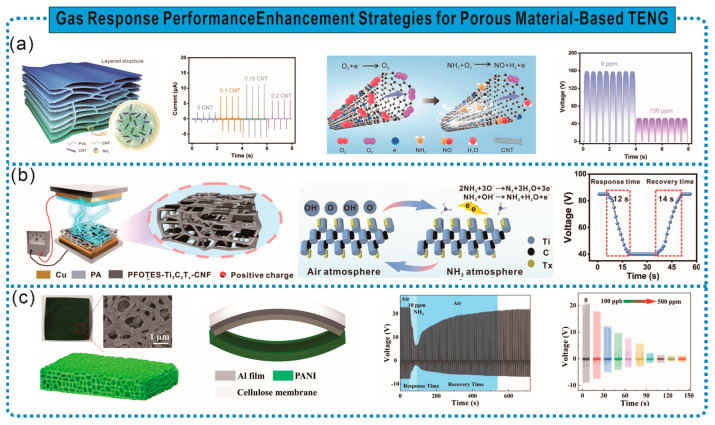
(**a**) Multi-stage porous friction electrogel realized by hetero-interface engineering [72]. (**b**) Gas-sensitive cellulose friction electric material for self-powered ammonia sensing [73]. (**c**) Rationally designed PANI/commercial cellulose paper-based TENG for high-sensitivity self-powered ammonia detection [74].

Frictional electric materials with gas sensitivity can achieve the concentration detection of specific gases in the environment; however, addressing issues such as material fragility, weak adsorption, low sensitivity, and a narrow detection range remains challenging. Research has found that these problems can be addressed through rational structural designs and the introduction of materials that exhibit specific reactions with gases. For instance, Gao et al. fabricated a cellulose-based frictional electric aerogel with a hierarchical porous structure composed of oxidized nanocellulose (TCNF), PVA, and CNTs through heterogeneous interface engineering. The combination of TCNF and CNTs formed hydrogen bond heterointerfaces, enhancing the interface stability and optimizing interface transmission. Moreover, the addition of CNTs increased the compressive deformation resistance of the aerogel by 40 times. Additionally, the high porosity (97.23%) of the aerogel provides more binding sites for gas molecules. TENG gas sensors based on this aerogel exhibit high sensitivity to ammonia gas, accurately identifying changes in the NH_3_ concentration within the range of 20–150 ppm, enabling the real-time wireless detection of food quality [72] (Figure 10a). Furthermore, introducing specific MOFs can enhance the adsorption capacity of gas-sensitive frictional electric materials. As shown in Figure 10b, Zhang et al. constructed a highly adsorbent gas-sensitive cellulose frictional electric material with a layered structure by adsorbing active silicon alcohol monomers onto CNF, and introducing Ti_3_C_2_Tx, followed by intermittent filtration and hot pressing drying. The presence of micro/nanoscale interfaces on the material surface and within the layered structure enhanced the connectivity between Ti_3_C_2_Tx nanosheets and provided more active sites for ammonia gas. The TENG based on this frictional electric material exhibits a rapid response/recovery (12 s/14 s), a high sensitivity response (V_air_/V_gas_ = 2.1), high selectivity response (37.6%), and low detection limit (10 ppm) to ammonia gas. Moreover, the TENG can accurately identify changes in the NH_3_ concentration within the range of 10–120 ppm and wirelessly transmit the signal to the user interface, facilitating the real-time online monitoring of ammonia gas in the environment [73].

Changes in the TENG output caused by chemical reactions between gases and specific functional groups can achieve precise gas detection, enhance detection sensitivity, and expand the detection range. For example, Yang et al. used commercial cellulose paper as a porous framework and synthesized PANI on it through in situ polymerization to prepare PANI/cellulose paper with a layered structure. Assembled with another nitrocellulose paper, it formed PANI/commercial cellulose paper-based TENG (PC-TENG) as a self-powered ammonia gas sensor. The reaction between PC-TENG and NH_3_ causes the protonation–deprotonation of PANI, resulting in changes in PANI’s surface friction state and impedance, thereby affecting the output of PC-TENG to achieve a response to NH_3_. PC-TENG exhibits excellent NH_3_-sensing characteristics, with a minimum detection limit as low as 100 ppb and a maximum detection limit of 500 ppm [74] (Figure 10c).

### 3.4. Environmental Tolerance Performance

The special structure of porous materials endows them with excellent moisture resistance, outstanding high-temperature resistance, good electromagnetic interference (EMI) shielding properties, and self-healing capabilities. Incorporating these characteristics into the design of the TENG provides new insights for expanding the development of TENG in emerging application fields and under special environmental conditions.

**Figure 11:**
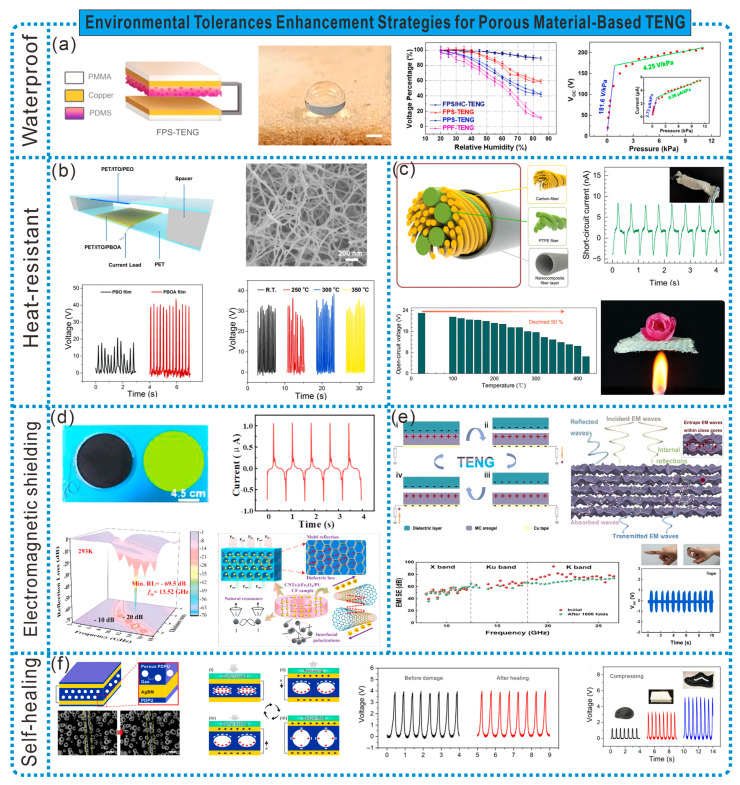
(**a**) Fluorinated polymer sponge-based TENG with superhydrophobicity [75]. (**b**) Polybenzazole aerogel-based TENG with excellent refractoriness and thermal stability [76]. (**c**) Aerogel nanocovered friction electric yarns to harvest electrical energy from high-temperature environments [77]. (**d**) Stretchable polyurethane composite foam-based TENG with tunable microwave absorption properties at high temperatures [78]. (**e**) Lightweight and flexible MXene/carboxymethyl cellulose aerogel for electromagnetic shielding, energy harvesting, and self-powered sensing [79]. (**f**) Self-healing viscous porous elastomers for gas–solid interaction power generation [80].

In humid environments, the presence of water reduces the generation of static charges on the working surface of the TENG, thereby affecting its output performance. This issue can be addressed by adopting surface hydrophobic modifications or gapless designs. For example, Peng et al. prepared an unencapsulated fluorinated polymer sponge-based friction nanogenerator (FPS-TENG) using a sugar-dissolving method and fluorination treatment. Thanks to the inherent hydrophobicity of the PDMS, porous structure, and low surface free energy of the fluorinated termination groups, FPS-TENG exhibited electrical outputs at 40% RH, with the output voltage being three times that of the pristine polymer film (PPF)-based TENG. The FPS-TENG assembled with hydrophobic copper electrodes maintained nearly 90% of its electrical output at 20–85% RH. Additionally, due to FPS’s excellent deformability, the FPS-TENG demonstrated high sensitivity of 181.6 V kPa^−1^ under low pressure conditions [75] (Figure 11a).

High temperatures can degrade the performance of electronic sensor components, even leading to the direct destruction of sensors. Therefore, it is necessary to prepare TENGs with excellent thermal insulation, flame resistance, and high-temperature resistance. As shown in Figure 11b, Qian et al. prepared heat-resistant TENGs by pairing poly(benzobisoxazole) aerogel (PBOA) as the negative friction material with different materials. The PBOA thin film has a high porosity of up to 94.5%, a specific surface area of 155–180 m^2^ g^−1^, and exhibits high strength, modulus, thermal resistance, and flame resistance. PBOA/PEO TENGs can serve as self-powered, highly sensitive sensors for monitoring human motion and detecting minor collisions. PBOA/Al TENGs demonstrate excellent performance in high-temperature environments (350 °C) [76]. Additionally, fabric-based TENGs with high-temperature resistance, thermal insulation, and flame retardancy can expand the application scope of smart wearable electronic fabric for the real-time monitoring of human motion and safety in extreme environments. As depicted in Figure 11c, Wang et al. fabricated multilayer stable silica aerogel nanoparticle-coated friction yarns using electrospinning and ancient twisting methods, exhibiting excellent high-temperature resistance and frictional electrical performance. These were manufactured into a yarn-based friction nanogenerator (Y-TENG) to harvest energy and sense biological movements at temperatures ranging from 25 to 400 °C. Smart protective suits made using Y-TENG can provide real-time sensing and rescue assistance for individuals working in high-risk environments [77].

Conventional TENG devices have relatively simple functions and are susceptible to various EMI in complex environments. Therefore, there is a need to develop TENGs that integrate effective energy harvesting with stable absorption and EMI shielding properties, achieving electromagnetic protection while collecting environmental mechanical energy. Here, Wen et al. developed a high-performance stretchable composite foam-based triboelectric nanogenerator (CF-TENG) with an adjustable microwave absorption (MA) capacity, using self-foaming PU as the elastic matrix, tadpole-shaped CNTs@Fe_3_O_4_ nanoparticles as the MA units, and conductive fillers. Benefiting from well-matched impedance, enhanced dielectric and magnetic losses, as well as the synergistic contributions of multiple reflections and scattering, the CF-TENG exhibited an excellent MA capability, demonstrating a strong absorption intensity of −68.5 dB at a thickness of 2.55 mm, with an effective frequency bandwidth of up to 2.55 GHz. Additionally, due to the temperature-dependent conductivity and adjustable porosity, the CF-TENG demonstrated a tunable MA, with the matching frequency shifting to higher frequency regions at elevated temperatures, widening the effective frequency bandwidth [78]. Conductive aerogels have been increasingly utilized in the field of EMI shielding for their ability to absorb electromagnetic radiation primarily, thus avoiding secondary radiation. As shown in Figure 11e, Cheng et al. prepared a TENG based on MXene Ti_3_C_2_Tx/CMC aerogel through a freeze-drying strategy, capable of both collecting biomechanical energy and shielding electromagnetic radiation. The TENG exhibited an excellent EMI shielding performance and energy harvesting capabilities, making it suitable for self-powered human health monitors to detect subtle biological movements (respiratory motions), thus protecting the human body from electromagnetic radiation while monitoring human health [79].

In the face of complex and harsh environments, frictional electric materials are inevitably prone to damage. Frictional electric materials with self-healing capabilities can sustain TENG’s output performance for longer periods, making it more adaptable to complex environments. As shown in Figure 11f, Xiong et al. prepared a supramolecular polysiloxane-dimethylethylenedioxime polyurethane (PDPU) porous elastomer with adhesion, air-tightness, and self-healing properties. Using this elastomer as the negative frictional electric material, they designed a gapless gas–solid interaction TENG (GS-TENG). The reversible dissociation and recombination of the urea–oxime–amino methyl carboxylate units endowed the PDPU elastomer with excellent elasticity and healability. A GS-TENG can serve as an independent wearable functional tactile skin for the self-powered sensing of touch pressure, human motion, and Parkinson’s gait. Furthermore, due to PDPU’s self-healing capability and outstanding elasticity, a GS-TENG is expected to regain its functionality after damage or extreme deformation [80].

### 3.5. Others

In addition to the above-mentioned characteristics, the diversity and designability of porous material structures also give them certain special functions. This enables some TENGs to possess specific functionalities, such as sound absorption, dust filtration, and flexible transparency. The introduction of these specific functionalities greatly expands the application environments and fields of TENGs.

Car tire noise is a common issue during driving, and with the development of autonomous driving vehicles, intelligent tires with self-powered sensing and noise reduction have become urgently needed. Kim et al. developed a friction nanogenerator (Tire-TENG) inside the tire using acoustic foam (AF) for autonomous driving and sound-absorbing intelligent tires. The Tire-TENG maintains the sound absorption capability of the original AF while utilizing the radial acceleration variation of the tire to generate electricity, achieving excellent noise reduction and high output power. Therefore, the Tire-TENG can provide semi-permanent power for internal intelligent sensor systems while achieving tire noise reduction [81].

Particulate matter (PM) serves as one of the primary pollutants in air pollution, posing threats to human life and health. Li et al. developed a frictional electric air filtration system based on a TENG using a layer of synthetic fiber filter cotton (or sponge) and a layer of a PTFE membrane. Due to the triboelectric effect and electrostatic attraction of the filter cotton and PTFE, the filtration efficiency of the filtering material for PM is significantly enhanced (20–40% increase in PM1.0 and PM2.5 filtration efficiency). Additionally, the positive charge generated by friction also greatly improves the interception efficiency of the filtering material for *Staphylococcus aureus* [82].

Flexible transparent friction nanogenerators have tremendous potential applications in self-powered biosensor systems, electronic skins, and wearable electronic devices. However, improving the output performance without compromising their optical properties poses challenges. He et al. prepared well-ordered nest-like porous PDMS films (NP-PDMS) using the hydrochloric acid etching of spherical ZnO nanoparticle aggregates, achieving good transparency (81.8%) due to the well-organized pores. They assembled it with transparent graphene electrodes to create a flexible transparent TENG (FT-TENG), achieving a high output performance without compromising the optical characteristics. The FT-TENG based on NP-PDMS film with a porosity of 12% exhibited a maximum output of 271 V and 7.8 μA, which were 3.7 times and 2.1 times higher than those of the TENG with flat PDMS films, respectively [83].

## 4. Sensing Applications

With the rapid development of the IoT and AI technology, self-powered sensors with portability, environmental adaptability, and reliability are receiving increasing attention. TENGs based on porous materials, characterized by flexibility, adjustability, high sensitivity, stability, and strong environmental adaptability, play a significant role in self-powered sensing, including pressure sensing, gas sensing, health monitoring, human–machine interactions, etc.

### 4.1. Pressure Sensing

Pressure sensors, as the medium for connecting the IoT and physical terminal information interactions, need to convey a large amount of tactile and pressure sensing information. In recent years, resistive [84,85], capacitive [86,87], and piezoelectric [88,89] pressure sensors have been widely used. However, resistive and capacitive pressure sensors require an external power source to operate, greatly limiting their applicability in extreme environments. When porous material-based TENGs are subjected to pressure, the capacitance formed between the material and the electrodes undergoes a change, thereby indicating a variation in pressure [90]. Although piezoelectric pressure sensors solve the problem of requiring an external power source, the types of materials that can be used as piezoelectric sensors are very limited, and they can only be applied in ordinary scenarios. Therefore, there is a need to develop pressure sensors with a self-powering capability, high sensitivity, and strong environmental adaptability.

**Figure 12:**
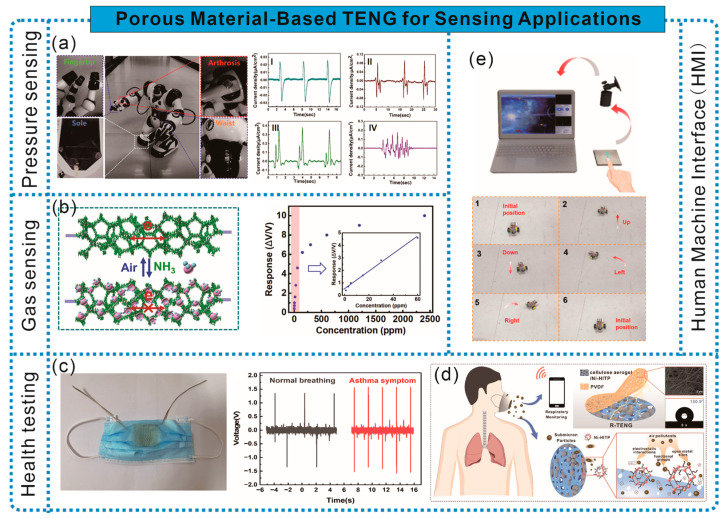
(**a**) UMS for motion recognition and machine control [91]. (**b**) ES-TENG as a self-powered sensor detecting toxic NH_3_ [93]. (**c**) TENG mask for detecting asthma symptoms [94]. (**d**) Self-powered air filter for respiratory monitoring and removal of submicron particles [95]. (**e**) SPTS device operating computer games and remotely controlling smart vehicles [96].

TENG-based pressure sensors, with highly sensitive, stable, environmentally adaptable, and self-powered characteristics, fully meet the requirements of the IoT’s development for pressure sensors [90]. TENGs based on porous materials not only have the advantages of miniaturization and being lightweight, but also exhibit excellent resilience, high sensitivity, and a good electrical output performance, making them highly suitable for designing highly sensitive frictional voltage pressure sensors. Moreover, due to the wide availability of porous materials and their design flexibility, TENGs based on porous materials can be designed in various styles to adapt to different application scenarios. For example, Zhao et al. designed an ultralight, self-powered, and adaptive motion sensor (UMS) using a composite material composed of graphene foam coated with ethyl cellulose and polystyrene microspheres dispersed in the foam pores. This UMS can detect the force, acceleration, and bending angle without being disturbed by an ambient temperature and humidity. The output current density of UMS is positively correlated with force, with an acceleration detection range of 0.1~1 m/s^2^ and a bending angle of 0~150°. When attached to different positions of the robot, it can be used for motion recognition and machine control (Figure 12a) [91].

### 4.2. Gas Sensing

Detecting specific gases in the ambient air can ensure environmental safety, human health, and food safety. However, traditional gas sensors heavily rely on external power sources during operation, limiting their further development in mobile monitoring and wearable sensing fields. TENGs, characterized by miniaturization, portability, and a self-powering capability, have rapidly developed in self-powered gas sensing applications, particularly showing outstanding potential in sensing and detecting gases such as ammonia, water vapor, and volatile organic compounds (VOCs) [92]. Porous materials provide more binding sites for gases due to their large surface area, and the advantages of easy modifications and a designable structure of porous materials enable TENG-based self-powered gas sensors to have higher sensitivity, a wider detection range, and a faster response time compared to traditional gas sensors. For instance, Liu et al. prepared an elastic TENG (ES-TENG) using a conductive elastic PU sponge modified with PANI nanowires for the self-powered sensing of toxic NH_3_. The self-powered sensor exhibited a wide NH_3_ concentration detection range (1–2400 ppm) and fast response (response time less than 3 s) [93] (Figure 12b). Additionally, Gao et al. developed a frictional electric gas sensor based on cellulose-based aerogels with a hierarchical porous structure, showing high sensitivity to ammonia. It accurately identified NH_3_ concentration changes within the range of 20–150 ppm, enabling the real-time wireless detection of food quality [72].

### 4.3. Health Monitoring

The state of our health is a matter of great concern for each of us. The real-time monitoring of our physical condition and the timely detection of issues can effectively safeguard our lives. Multifunctional TENG-based self-powered sensors play a significant role in monitoring human health, such as in respiratory monitoring (masks) and sleep tracking (pillows).

Breathing reflects multiple aspects of our body, and the real-time monitoring of one’s respiratory status can help us detect abnormal situations and respond promptly. Moreover, TENG devices used for detection must have good biocompatibility and wearability to meet practical needs. As shown in Figure 12c, Tan et al. designed a bioporous TENG mask based on a silk fibroin@MXene composite aerogel and PDMS sponge, which has excellent breathability and can detect the respiratory status based on frequency changes. It can be used as a real-time asthma detector to diagnose asthma symptoms [94]. Additionally, Fu et al. designed a self-powered air filter based on a cellulose aerogel-based TENG, which can filter submicron particles and monitor the wearer’s respiratory status in real-time based on frequency and intensity variations [95] (Figure 12d). Sleep monitoring is also an important aspect of assessing our physical condition, and monitoring sleep quality helps us gain deeper insights into our physical condition. Kou et al. developed a smart pillow based on TENGs, which can record real-time head movements during sleep and provide early warnings for emergencies [26].

### 4.4. Human–Computer Interactions

TENGs not only assist and enhances human capabilities through sensing and actuation, but also wearable TENGs can serve as intermediaries between humans and machines, thus fostering the development of human–computer interactions. As a medium for human–computer interactions, TENGs can facilitate autonomous and biocompatible interactions by utilizing inherent human information, thus overcoming the issue of complex interfaces in traditional human–computer interactions. For instance, Doganay et al. developed a self-powered electronic wristband based on fabric-based TENGs that can serve as a human–computer interaction interface for controlling simple computer operations [48]. Additionally, multifunctional self-powered tactile sensors play a significant role in the development of intelligent human–machine interaction (HMI) systems. Zhang et al. have demonstrated that the integration of self-powered sensors of TENGs with machine learning methods can further achieve highly accurate and intelligent human–computer interactions [96]. Su et al. prepared a hybrid self-powered porous structure tactile sensor (SPTS) by integrating a single-piece multifunctional friction-induced electroluminescence module and a single-electrode TENG. The programmable optoelectronic dual-mode human–machine interface system established based on SPTS can remotely control smart vehicles and operate computer games by recognizing finger touch trajectories (Figure 12e) [97].

## 5. Summary and Outlook

Porous materials have a wide range of sources, including conductive and non-conductive materials. They can be designed as electrodes or friction layer materials for TENGs. Porous materials have various advantages, including a large specific surface area, low density, excellent mechanical elasticity (such as flexibility, stretchability, and compressibility), breathability, low thermal conductivity, and good biocompatibility, etc. With these advantages, porous electrodes and friction materials have received extensive attention from researchers in recent years, defining them as the materials for the next generation of TENGs and promoting the development of TENGs in the field of self-powered smart sensing. There is a diversity of porous materials, and here we mainly analyze the structural forms and construction methods of porous materials in existing TENGs, including aerogels, foam sponges, electrospinning, 3D printing, and fabric structures. The comprehensive performance of porous material-based TENGs depends on the selection of materials, the design of porous structures, and the assembly of devices. Therefore, we also emphasize the design strategies of porous materials in improving the TENG performance, including the mechanical performance, electrical performance, gas responsiveness, and environmental durability. Finally, we introduce the applications of porous material-based TENGs in self-powered sensing aspects such as pressure sensing, health monitoring, and human–machine interactions.

Porous materials, with their numerous advantages, have become the ideal choice for TENG electrodes and friction materials. Porosity is an important factor affecting the performance of TENGs based on porous materials. By changing the material or processing factors, various forms of pores with different shapes and sizes can be achieved, and the frictional electrical performance of TENGs can be improved by optimizing the design of pores. Although the influence of porosity on the frictional electrical performance of porous material-based TENGs has been studied, research exploring the effects of other characteristics (such as the pore size distribution) on the TENG’s performance is lacking. In addition, the structural design of porous material-based TENGs is also a major aspect affecting their frictional electrical performance. For example, an early TENG typically required additional air gaps between two friction layers to complete the contact-separation process. However, the design of additional gaps will affect the durability and lifespan of the TENG. Subsequently, a gapless TENG based on the inherent air gaps of porous materials was designed to solve this problem. However, the frictional electrical performance and working mode of gapless TENGs are limited, so how to reconcile these issues is also a problem that needs to be addressed in the future. Therefore, there is still considerable room for improvement in the frictional electrical performance, and research in this area needs to be strengthened in the future. Moreover, certain specific properties such as high temperature and humidity resistance, flexibility, and high stretchability can be achieved through composites with other functional materials. However, ensuring that other properties of TENGs are not affected while meeting the requirements of complex and customized electrode structures is also a challenge. Furthermore, biomimetic porous structures inspired by nature provide new strategies for designing high-performance porous materials.

In the future, with the deepening development of AI and HMI, the Metaverse, as an emerging concept, will receive widespread attention in academia. It serves as a set of interconnected 3D virtual spaces that reflect the real world, providing users with immersive interactive, collaborative, and social activity environments. Of course, this also implies new demands for the multifunctional integration of wearable self-powered sensors. In brief, despite challenges that still exist, the rapid development and widespread application trends of TENGs based on porous materials are unstoppable in the high-tech era.
